# Higher Concentrations of Essential Trace Elements in Women Undergoing IVF May Be Associated with Poor Reproductive Outcomes Following Single Euploid Embryo Transfer

**DOI:** 10.3390/cells13100839

**Published:** 2024-05-15

**Authors:** Roberto Gonzalez-Martin, Andrea Palomar, Silvia Perez-Deben, Stefania Salsano, Alicia Quiñonero, Laura Caracena, Rocio Fernandez-Saavedra, Rodolfo Fernandez-Martinez, Estefania Conde-Vilda, Alberto J. Quejido, Juan Giles, Carmen Vidal, Jose Bellver, Francisco Dominguez

**Affiliations:** 1 IVI-RMA Global Research Alliance, IVI Foundation, Instituto de Investigación Sanitaria La Fe (IIS La Fe), 46026 Valencia, Spain; roberto.gonzalez@ivirma.com (R.G.-M.); andrea.palomar@ivirma.com (A.P.); alicia.quinonero@ivirma.com (A.Q.); laura.caracena@ivirma.com (L.C.); juan.giles@ivirma.com (J.G.); carmina.vidal@ivirma.com (C.V.); jose.bellver@ivirma.com (J.B.); 2 IVI-RMA Global Research Alliance, IVI-RMA Valencia, 46015 Valencia, Spain; 3 Unit of Mass Spectrometry and Geochemical Applications, Chemistry Division, Department of Technology, Centro de Investigaciones Energéticas, Medioambientales y Tecnológicas (CIEMAT), 28040 Madrid, Spain; rocio.fernandez@ciemat.es (R.F.-S.); rodolfo.fernandez@ciemat.es (R.F.-M.); estefania.conde@ciemat.es (E.C.-V.); alberto.quejido@ciemat.es (A.J.Q.); 4 Department of Pediatrics, Obstetrics and Gynecology, Faculty of Medicine of Valencia, 46010 Valencia, Spain

**Keywords:** essential trace elements, biofluids, ovarian response, IVF outcomes, ICP-MS

## Abstract

Essential trace elements are micronutrients whose deficiency has been associated with altered fertility and/or adverse pregnancy outcomes, while surplus may be toxic. The concentrations of eight essential trace elements were measured using inductively coupled mass spectrometry (ICP-MS) and assessed with respect to clinical in vitro fertilization (IVF) outcomes in a population of 51 women undergoing IVF with intracytoplasmic sperm injection (ICSI), pre-implantation genetic screening for aneuploidy (PGT-A), and single frozen euploid embryo transfer (SET/FET). Specifically, copper (Cu), zinc (Zn), molybdenum, selenium, lithium, iron, chromium, and manganese were quantified in follicular fluid and whole blood collected the day of vaginal oocyte retrieval (VOR) and in urine collected the day of VOR and embryo transfer. We found that the whole blood Cu/Zn ratio was significantly associated with superior responses to ovarian stimulation. Conversely, the whole blood zinc and selenium concentrations were significantly associated with poor ovarian response outcomes. Higher levels of whole blood zinc and selenium, urinary selenium, lithium, and iron had significant negative associations with embryologic outcomes following IVF. Regarding clinical IVF outcomes, higher urinary molybdenum concentrations the day of VOR were associated with significantly lower odds of implantation and live birth, while higher urinary Cu/Mo ratios on the day of VOR were associated with significantly higher odds of implantation, clinical pregnancy, and live birth. Our results suggest that essential trace element levels may directly influence the IVF outcomes of Spanish patients, with selenium and molybdenum exerting negative effects and copper-related ratios exerting positive effects. Additional studies are warranted to confirm these relationships in other human populations.

## 1. Introduction

At trace concentrations, certain elements such as copper (Cu), zinc (Zn), molybdenum (Mo), selenium (Se), lithium (Li), iron (Fe), chromium (Cr), and manganese (Mn) are essential for the proper functioning of living organisms. Trace elements are tightly regulated to maintain homeostasis, availability, and various biological functions within the human body, including those related to fertility [1,2,3,4]. The general population is primarily exposed to these elements through diet [2,3]. Despite both excessive and inadequate exposure to these compounds being associated with adverse health effects [2,3], the majority of existing research is focused on conditions related to trace element deficiencies [3].

Some subfertile women present low concentrations of essential trace elements [2,5,6,7,8]. In these cases, supplementation may help mitigate associated conditions and improve reproductive outcomes [5,7], but the pathogenesis of reproductive disorders driven by irregular trace element concentrations remains unclear. The reported associations between essential trace element levels and assisted reproductive treatment (ART) outcomes in cohorts of American women undergoing in vitro fertilization (IVF) are inconsistent between studies [9,10,11,12]. These discrepancies may be due to geographical and cultural factors or variable ART methodologies between clinics.

Thus, the aim of this study was to determine the impacts of essential trace element concentrations in follicular fluid (FF), whole blood (collected on the day of vaginal oocyte retrieval [VOR]), and urine (collected on the embryo transfer day) on the IVF outcomes of women undergoing intracytoplasmic sperm injection (ICSI) and pre-implantation genetic screening for aneuploidy (PGT-A) prior to single frozen euploid embryo transfer (SET/FET).

## 2. Materials and Methods

### 2.1. Study Population

Fifty-one women (aged 18–42) undergoing ICSI with PGT-A and SET/FET between September 2018 and November 2019 at IVI-RMA Valencia (Spain) were enrolled in this prospective, single-center pilot study. Participants were excluded if they had severe male factor infertility, untreated systemic or endocrine disorders, abnormal karyotypes, thrombophilia, an atypical uterus, an irregular endometrial pattern, or endometrial thickness measuring less than 7 mm on the embryo transfer day. Those who met the criteria received the same standard clinical and laboratory care they would have if they had not taken part in the study.

### 2.2. Collection of Follicular Fluid, Whole Blood, and Urine Samples

Urine was collected from fasting participants the morning of VOR (n = 50) and embryo transfer (ET; n = 27) in sterile polypropylene containers, and then stored at 4 °C. Urine samples underwent centrifugation at 500× *g* for 7 min to separate the sediment and collect the supernatant (urine), which was aliquoted, and then stored at −80 °C. Blood samples (n = 40) collected from fasting participants on the day of VOR were drawn into EDTA tubes, aliquoted, and stored at −80 °C. After VOR, the cumulus–oocyte complexes were isolated for clinical use. The FF aspirated from each patient was pooled, centrifuged at 1000× *g* for 3 min to remove cellular debris, aliquoted, and stored at −80 °C. Trace element-free plastic materials were used for sample processing and storage. A set of blanks were prepared and stored in the same way as the different biological fluids to monitor possible contamination. From the 51 participants, 50 urine samples from the morning of VOR, 27 urine samples from the morning of ET, 29 FF samples, and 40 whole blood samples could be collected. All samples were shipped on dry ice, first to the IVI Foundation (Valencia, Spain) for processing, and then to the Mass Spectrometry and Geochemical Applications Unit of the CIEMAT (Madrid, Spain) for the quantification of essential trace elements. Upon arrival at the CIEMAT, the samples were stored at −80 °C until further analysis.

### 2.3. Quantification of Essential Trace Elements by ICP-MS

The concentrations of the essential trace elements in FF, whole blood, and urine were assessed using inductively coupled plasma mass spectrometry (ICP-MS) by employing an i-CapRQ mass spectrometer (Thermo Fisher Scientific, Madrid, Spain) equipped with a quadrupole analyzer and dual mode secondary electron multiplier (SEM) as a detection system. Except for lithium, all trace elements were analyzed using a collision cell (CCT) operating in kinetic energy discrimination (KED) mode to mitigate polyatomic interferences from the biological matrices.

The day before ICP-MS, samples were thawed and refrigerated at 4 °C. To prepare samples for ICP-MS, 0.5 mL of each FF sample was diluted to a ratio of 1:20 in 0.5% (*v*/*v*) HNO_3_ (distilled in situ using a DuoPUR Sub-boiling Distillation System [Milestone Inc., Madrid, Spain]) and 0.0005% (*v*/*v*) Triton^®^ X-100 (Sigma-Aldrich, Madrid, Spain) as a surfactant. Internal standards included 1 μg/L of gallium (Ga), indium (In), and lutetium (Lu) (SPEX CertiPrep, Thermo Fisher Scientific, Madrid, Spain and Inorganic Ventures, Isostandards, Madrid, Spain). Alternatively, 0.5 mL of each whole blood sample was digested in a DigiPrep block (SCP SCIENCE, Baie-d’Urfé, QC, Canada) with temperature ramping by using 2 mL of 65% HNO_3_ and 0.1 mL of 40% (*w*/*v*) Suprapur^®^ hydrofluoric acid (Merck Millipore, Madrid, Spain) at 75 °C for 15 min, followed by 1 mL of 30% (*v*/*v*) H_2_O_2_ (Merck Millipore, Madrid, Spain) at 115 °C for 60 min. Following the addition of the internal standards (SPEX CertiPrep, Thermo Fisher Scientific, Madrid, Spain and Inorganic Ventures, Isostandards, Madrid, Spain), digested whole blood samples were adjusted to 10 mL with Milli-Q water (18.2 MΩ/cm; Merck Millipore, Madrid, Spain). Finally, 0.5 mL of each urine sample was diluted to a ratio of 1:10 in 2% (*v*/*v*) HNO_3_ with 1 μg/L of Ga, In, and Lu (SPEX CertiPrep, Thermo Fisher Scientific, Madrid, Spain and Inorganic Ventures, Isostandards, Madrid, Spain).

Blanks and calibration curves (serial dilutions ranging from 0.01 to 500 µg/L) (SPEX CertiPrep, Thermo Fisher Scientific, Madrid, Spain and Inorganic Ventures, Isostandards, Madrid, Spain) were prepared daily in 2% (*v*/*v*) HNO_3_ for whole blood and urine samples or 0.5% (*v*/*v*) HNO_3_ and 0.0005% (*v*/*v*) Triton^®^ X-100 for FF samples. Both types of standards were supplemented with internal standards (Ga, In, and Lu), each at a final concentration of 1 µg/L.

To account for the dilution of essential trace elements in urine, we normalized our results to the levels of creatinine. The creatine was measured in the aliquoted urine, which previously stored at −80 °C, using a commercially available kit (Creatinine Parameter Assay Kit, KGE005, Bio-Techne R&D Systems, Madrid, Spain).

### 2.4. Clinical Management and Outcome Assessment

Baseline characteristics of patients, including their dates of birth, weights, and heights, were collected upon enrollment to calculate age and body mass index (BMI; kg/m^2^). Additional demographic variables, such as race/ethnicity, education, and smoking status, were self-reported through a questionnaire. Serum estradiol (E2) levels on the ovulation trigger day were measured using an in-clinic automated electrochemiluminescence immunoassay.

All patients underwent COS using a gonadotropin releasing hormone (GnRH) antagonist protocol. The dosage of gonadotropins was determined based on the clinician’s assessment of ovarian reserve. When follicles reached a diameter of 15–20 mm, final oocyte maturation was induced by administering human chorionic gonadotropin (hCG) and/or a GnRH agonist trigger, followed by ultrasound-guided VOR 36 h later [13,14,15].

Cumulus cells were removed from the oocytes, and the number and maturation of recovered oocytes were recorded. ICSI was performed to minimize the risk of DNA contamination during PGT-A and to standardize fertilization procedures. The number of fertilized oocytes was assessed 18 h after ICSI, and sequential culture medium was used to culture embryos to the blastocyst stage [13,14,15]. Trophectoderm biopsies for PGT-A were obtained before embryo vitrification [13,14,15].

A single euploid embryo was transferred following endometrial priming with oral estrogen and intramuscular progesterone [13,14,15]. Embryos were thawed and transferred into the uterine cavity using a catheter under ultrasound guidance on the day of substitution.

Internal protocols for evaluating clinical IVF outcomes were followed. Implantation was defined by a serum hCG concentration >6 mIU/mL approximately 14 days post-embryo transfer. Clinical pregnancies were confirmed through ultrasonography, and live births were defined as neonates born after 24 weeks of gestation.

### 2.5. Statistical Analysis

We conducted all statistical analyses using R software (version 3.6.2). Participants’ baseline demographic and reproductive characteristics were presented as median ± interquartile ranges (IQRs) or percentages, all summarized using the “tableone” package [16]. Correlation matrices were generated with the “corrplot” package [17] to examine the relationships among distinct essential trace elements within and between each biofluid. To assess the association between essential trace element concentrations and IVF outcomes, we employed multivariable generalized linear models. Mean differences for trigger day E2 concentrations were treated as a continuous variable and estimated with a Gaussian distribution. Discrete count variables, such as the total number of retrieved oocytes, relative proportion of mature (metaphase II; MII) oocytes (offset by the total number of oocytes retrieved), relative proportion of fertilized embryos (offset by the total number of MII oocytes), relative proportion of blastocysts (offset by the total number of fertilized embryos), and relative proportion of euploid embryos (offset by the total number of blastocysts), were calculated using Poisson distributions. Essential trace element concentrations were modeled as continuous variables (log-transformed), and linear associations were obtained by comparing the increase between the 20th and 80th percentile. Furthermore, the odds ratios (ORs) for the probability of reaching embryo transfer, clinical IVF outcomes (i.e., implantation, clinical pregnancy, and live birth) relative to embryo transfers, as well as the reproductive goal (probability of live birth for each initiated cycle) were calculated using a binomial distribution with the “questionr” package [18].

To facilitate interpretation of the results, we present the marginal population means adjusted for all covariates in the model. Potential confounders included factors that were previously associated with reproductive outcomes and exposure to essential trace elements [19]. The final models were adjusted for age (as a continuous variable), BMI (as a continuous variable), and smoking status (categorized as never, former, or current). In all cases, *p* < 0.05 was considered statistically significant.

## 3. Results

### 3.1. Baseline Characteristics

Among the fifty-one participants, the median age and BMI were 39 years [IQR: 38, 41] and 22.97 kg/m^2^ [IQR: 20.63, 25.12], respectively; 94.1% of the participants were Hispanic–White, 71% had a university degree, and 50% had never smoked (Table 1). All participants reported a similar dietary pattern, and none of the participants indicated any nutritional supplementation.

The median total dose of follicle stimulating hormone (FSH) plus human menopausal gonadotropin (hMG) administered during controlled ovarian stimulation (COS) was 3300.00 IU [IQR: 2437.50, 3925.00], and the trigger day E2 concentration was 2083.00 pg/mL [IQR: 1622.50, 4045.50] (Table 1).

From each participant, there was a median yield of 12.00 oocytes [IQR: 7.00, 16.00], of which 80 ± 17% were in metaphase II (MII). The fertilization, blastulation, and euploidy rates were, respectively, 75 ± 25%, 54 ± 28%, and 42 ± 34%. Among the 70.6% (36/51) of participants who underwent an embryo transfer, the implantation rate was 63.9%, the clinical pregnancy rate was 52.8%, and the live birth rate was 47.2%. Of the 51 participants, 33.3% achieved their goal of having a live newborn (Table 1).

### 3.2. Essential Trace Element Distribution among Biofluids

Table 2 indicates the proportion of samples with detectable levels of essential trace elements and the distributions of their concentrations in FF, whole blood, and urine collected during or on the day of VOR and in urine collected on the embryo transfer day. Essential trace elements that were below the quantification limit in >50% of the samples were excluded from subsequent analyses (Table 2).

In general, there were no correlations among the essential trace element concentrations in FF, blood, and both types of urine samples (Figure S1). However, there were significant relationships between the essential trace element concentrations within each biological fluid. Specifically, in FF, there were strong positive correlations between selenium and copper (r = 0.74) and zinc (r = 0.79), and there was a strong negative correlation between molybdenum and the Cu/Mo ratio (r = −0.86). In whole blood, copper had a strong positive correlation with the Cu/Mo ratio (r = 0.74), and molybdenum had a strong positive correlation with lithium (r = 0.76). In urine from the day of VOR, copper had a strong positive correlation with the Cu/Zn ratio (r = 0.71), and zinc had a strong negative correlation with the Cu/Zn ratio (r = −0.78). Finally, in urine collected on the day of embryo transfer, copper had a strong positive correlation with the Cu/Mo ratio (r = 0.76) (Figure S2).

### 3.3. Associations between Essential Trace Element Concentrations and Ovarian Response Outcomes

The associations between the log-transformed trace element concentrations and ovarian response outcomes were evaluated using multivariate models adjusted for age, BMI, and smoking status. The data are presented as the mean differences with relative proportions (95% CI) between the 20th and 80th percentiles.

**Figure 1:**
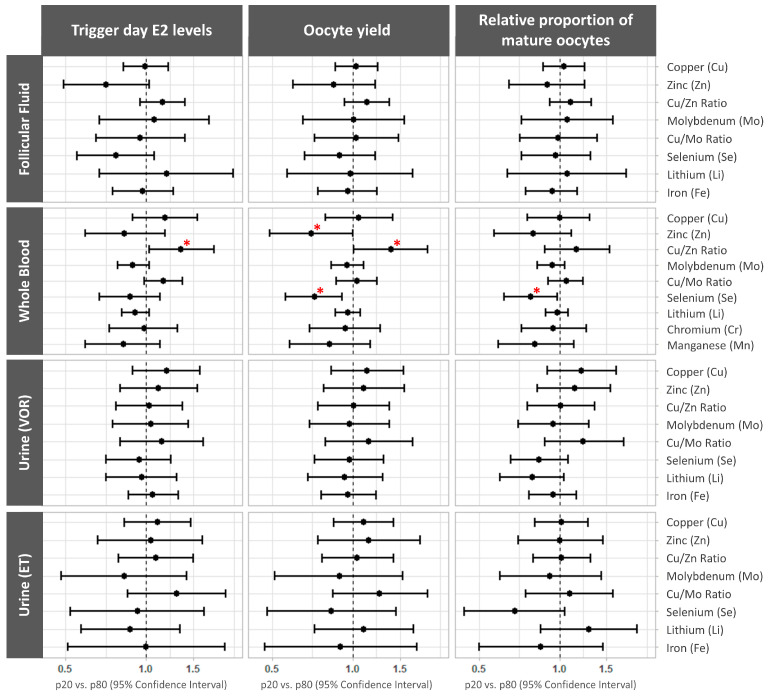
Forest plots representing the mean differences (95% confidence interval) in ovarian response-related outcomes by essential trace element concentrations. The mean differences (95% CI) are presented for the trigger day estradiol (E2) levels, the total number of retrieved oocytes, and the relative proportion of mature (MII) oocytes across the essential trace elements quantified in each biofluid. The data were adjusted for age (continuous), BMI (continuous), and smoking status (never, former, or current). * *p* < 0.05. ET, embryo transfer; VOR, vaginal oocyte retrieval.

The whole blood Cu/Zn ratio was significantly associated with the trigger day E2 levels (p20 vs. p80 (95% CI): 1.35 (1.03, 1.79), *p* trend = 0.033) and a greater oocyte yield (p20 vs. p80 (95% CI): 1.39 (1.01, 1.91), *p* trend = 0.042). In contrast, a significant negative association was observed between whole blood zinc and selenium concentrations and the oocyte yield (p20 vs. p80 (95% CI): 0.70 (0.49, 1.00), *p* trend = 0.047; and 0.72 (0.56, 0.91), *p* trend = 0.009; respectively). The whole blood selenium concentrations were also negatively correlated with the relative proportion of MII oocytes (p20 vs. p80 (95% CI): 0.78 (0.62, 0.98), *p* trend = 0.031) (Figure 1, Table S1).

In this population, there were no statistically significant associations between the remaining trace elements and biological matrices.

### 3.4. Association between Essential Trace Element Concentrations and Preimplantation IVF Outcomes

The associations between the log-transformed trace element concentrations and preimplantation IVF outcomes were also assessed using multivariable models adjusted for age, BMI, and smoking status. The data are presented as the mean differences with the relative proportions (95% CI) between the 20th and 80th percentiles.

**Figure 2:**
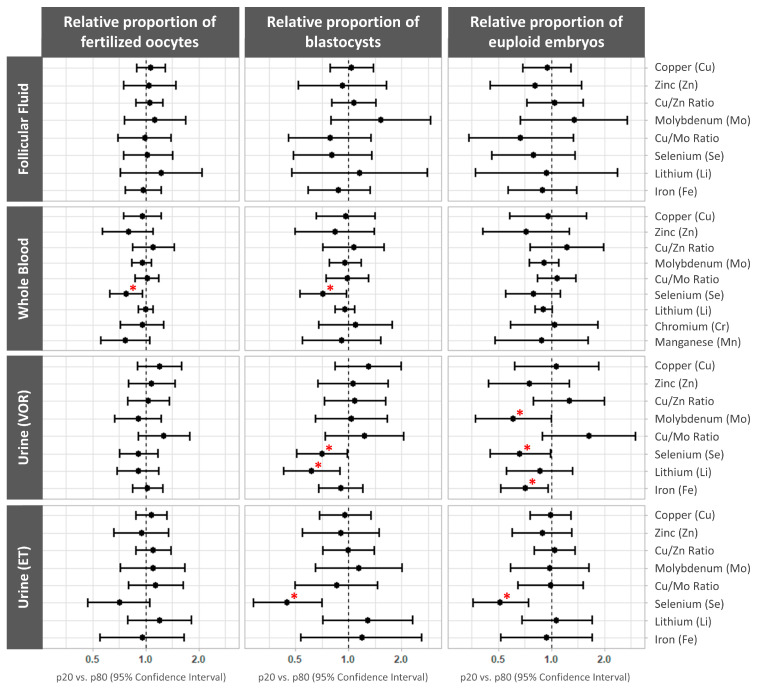
Forest plots representing the mean differences (95% confidence interval) in the preimplantation IVF outcomes by essential trace element concentrations. The mean differences (95% CI) for the relative proportions of mature (MII) oocytes that successfully fertilized, embryos developing to the blastocyst stage, and euploid blastocysts across the essential trace elements were quantified in each biofluid. The data were adjusted for age (continuous), BMI (continuous), and smoking status (never, former, or current). * *p* < 0.05. ET, embryo transfer; VOR, vaginal oocyte retrieval.

The whole blood selenium levels were negatively associated with the relative proportions of fertilized oocytes and blastocysts (p20 vs. p80 (95% CI): 0.78 (0.63, 0.96), *p* trend = 0.02, and 0.72 (0.53, 0.98), *p* trend = 0.04; respectively). Similarly, the urinary selenium and lithium levels on the day of VOR were negatively associated with the relative proportion of fertilized embryos (p20 vs. p80 (95% CI): 0.71 (0.51, 0.99), *p* trend = 0.043; and 0.62 (0.43, 0.90), *p* trend = 0.013; respectively), while the urinary molybdenum, selenium, and iron concentrations on the day of VOR were negatively associated with the relative proportion of euploid embryos (p20 vs. p80 (95% CI): 0.61 (0.37, 1.00), *p* trend = 0.05; 0.66 (0.45, 0.99), *p* trend = 0.045; and 0.71 (0.52, 0.96), *p* trend = 0.03; respectively). Finally, the urinary selenium levels the morning of ET were negatively associated with the relative proportions of blastocysts and euploid embryos (p20 vs. p80 (95% CI): 0.45 (0.29, 0.71), *p* trend = 0.002; and 0.51 (0.36, 0.74), *p* trend = 0.001; respectively) (Figure 2, Table S2).

### 3.5. Association between Essential Trace Element Concentrations and Clinical IVF Outcomes

The associations between the essential trace element concentrations in FF, blood, and urine collected the morning of VOR and in urine collected on the day of embryo transfer and clinical IVF outcomes were assessed using multivariable models adjusted for age, BMI, and smoking status. The data are presented as odds ratios (95% CI).

**Figure 3:**
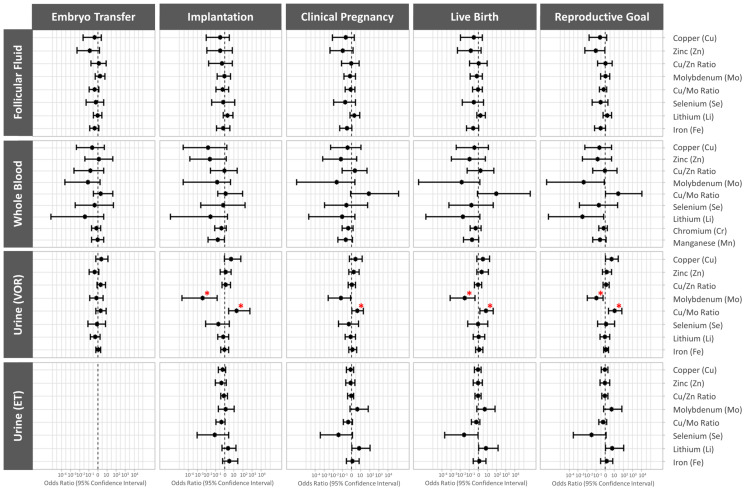
Forest plot representing the odds ratios (95% confidence interval) for the clinical reproductive outcomes. The odds ratios (95% CI) are presented for embryo transfer, implantation, clinical pregnancy, live birth, and reproductive goal (achievement of a live birth in a given cycle) following single frozen euploid embryo transfer across the essential trace elements quantified in each biofluid. The data were adjusted for age (continuous), BMI (continuous), and smoking status (never, former, or current). * *p* < 0.05. ET, embryo transfer; VOR, vaginal oocyte retrieval.

Higher urinary molybdenum levels the morning of VOR were associated with significantly lower odds of implantation (OR (95% CI): 0.004 (0.00002, 0.17), *p* value = 0.014), live birth (OR (95% CI): 0.03 (0.0008, 0.48), *p* value = 0.036), and the patients’ reproductive goals being achieved (OR (95% CI): 0.1 (0.01, 0.62), *p* value = 0.023). Conversely, higher urinary Cu/Mo ratios the morning of VOR were associated with significantly higher odds of implantation (OR (95% CI): 23.51 (3.04, 689.52), *p* value = 0.016), clinical pregnancy (OR (95% CI): 4.55 (1.23, 22.67), *p* value = 0.037), live birth (OR (95% CI): 6.96 (1.65, 44.38), *p* value = 0.018), and the patients’ reproductive goals being achieved (OR (95% CI): 10.5 (2.49, 69.61), *p* value = 0.005) (Figure 3, Table S3).

## 4. Discussion

The aim of this study was to investigate whether the levels of eight essential trace elements in three biological matrices on the day of VOR or embryo transfer were related to ART outcomes in Spanish women undergoing ICSI and in those undergoing SET/FET with PGT-A. Our study design accounted for the confounding factors of IVF treatment: selecting ICSI cycles standardizes fertilization procedures; FET cycles mitigate the potential deleterious effects of COS; and PGT-A avoids possible implantation failures derived from aneuploidy.

The associations between essential trace element exposure and IVF treatment outcomes have shown to be inconsistent across prior studies [9,10,11,12,20,21,22]. The discrepancies may reflect variations in ART protocols and in demographic, geographic, and sociocultural exposure factors.

We previously evaluated whether the reproductive outcomes of American women undergoing similar IVF treatment with ICSI, PGT-A, and SET-FET were related to essential trace element levels. Unfortunately, the distinct socio-demographic and lifestyle characteristics of the American and Spanish cohorts and their significantly different reproductive outcomes hinder comparison. On average, the Spanish participants had lower BMI levels, were older, predominantly Hispanic–White, and tobacco consumers, with 19.6% being active smokers. Older patients who smoked tended to have inferior reproductive outcomes, which unfortunately made it difficult to pool the results from both populations and draw combined conclusions.

In the American patients undergoing IVF, the FF levels of essential trace elements were positively correlated with those in plasma [12]. However, in the Spanish patients undergoing IVF, we did not observe positive correlations between biofluids, which was likely due to the analysis of whole blood rather than plasma. Therefore, biofluid selection should be accounted for in future studies. Furthermore, FF was not a good predictor of IVF outcomes in the Spanish patients, which contrasts what we observed in our previous study [12]. Given that the unadjusted models demonstrated that the Cu/Zn ratio in FF was positively associated with the number of mature oocytes recovered, and higher FF molybdenum levels were related to blastocyst development, it is possible that the FF sample size was too small to accurately predict IVF outcomes in the Spanish cohort.

In this study, we found that higher whole blood and urinary selenium levels were related to poor ovarian stimulation responses and preimplantation IVF outcomes. On the other hand, the Cu/Zn and Cu/Mo ratios, but not the copper levels on their own, were related to positive outcomes in both the Spanish and American cohorts. Furthermore, overall, the effects of lithium appeared to be less detrimental in the Spanish cohort than in the American one. Finally, the urinary molybdenum levels the morning of VOR were negatively correlated with clinical IVF outcomes in both the Spanish and American groups.

Selenium is a metalloid that is essential for humans because it is a structural constituent of the amino acid selenocysteine [3]. This amino acid is a fundamental component of selenoproteins, a family of 25 proteins with key roles in antioxidant defenses [2,3]. The main route of selenium exposure in the general population is diet. Selenium is found in seafood, organ meats, nuts, grains, cereals, and dietary supplements [2,3]. Although smoking was associated with a lower selenium body burden [3], smoking status did not relate to selenium levels in our cohort.

The whole blood selenium concentration correlated well with dietary selenium intake and was found to be a good biomarker because selenium has a half-life of two months [3]. Selenium is mainly excreted through urine, and its methylation status depends on the selenium body burden [3].

Selenium deficiency is associated with reproductive and obstetric complications, such as female and male infertility, pregnancy loss, pre-eclampsia, preterm delivery, and gestational diabetes [7,23,24]. However, the relationship between selenium concentrations and reproductive outcomes following IVF/ET remains unclear. Some authors reported that higher serum and hair selenium concentrations are associated with better responses to ovarian stimulation [25,26], while others did not observe these associations for serum nor FF selenium [27]. In American women undergoing IVF, we observed that higher plasma selenium levels were associated with lower trigger day E2 levels [12]. In the Spanish patients undergoing IVF, the statistical models adjusted for age, BMI, and smoking status revealed that higher whole blood selenium levels were associated with poor ovarian stimulation responses, impaired fertilization, and blastocyst development, while higher urinary selenium levels (VOR and ET) were associated with impaired blastocyst development and fewer euploid embryos. These observations align with the lower blastulation rates accompanying high FF selenium concentrations [10,20,27].

Despite being an essential trace element, the selenium exposure response curve is U-shaped with a narrow margin of safety [3,28]. Selenium supplementation can be beneficial for deficient individuals, but excessive selenium exposure may cause adverse health effects (e.g., male infertility) [3,29,30]. Thus, copious selenium supplementation is not recommended for individuals with adequate selenium levels [23].

Molybdenum functions as a cofactor of several metalloenzymes with oxidoreductase activity [2,31]. These enzymes help metabolize drugs, toxins, nitrogenous compounds (e.g., nucleotides and sulfur amino acids), and aromatic aldehydes [2,31]. The main sources of dietary molybdenum include leafy vegetables, grains, seeds, legumes, animal organs, and milk [2,3]. Once absorbed in the gastrointestinal tract, molybdenum enters the bloodstream and accumulates in the liver and kidneys. Seeing as approximately 90% of absorbed molybdenum is eliminated through renal excretion [3,31], urinary molybdenum is a highly sensitive biomarker of recent molybdenum intake [3]. Nevertheless, it should be noted that the presence of copper and sulfates accelerates molybdenum elimination by inhibiting tubular reabsorption [3,31].

Similar to our previous American cohort study, which identified a negative association between higher urinary molybdenum the morning of VOR and clinical IVF outcomes [12], Spanish patients undergoing IVF with higher urinary molybdenum the morning of VOR produced significantly fewer euploid embryos and had lower odds of successfully achieving implantation, live birth, and their reproductive goals. Given the consistent observations in both populations, further studies are warranted to discern the mechanisms of molybdenum in human female reproductive tissues.

Ingesting high doses of molybdenum led to altered estrous activity and impaired embryogenesis in mice [32]. The hypothesis that fertility declines due to molybdenum competing with and dysregulating copper cofactor enzymes [32] was supported by other reports of excessive molybdenum consumption inducing copper deficiency [31,33], whereas high copper concentrations protect against molybdenum toxicity [34,35]. Our study corroborated these findings, with higher Cu/Mo ratios in urine on the day of VOR being associated with superior odds of implantation, clinical pregnancy, live birth, and reproductive success. Conversely, in our previous American cohort study, we observed that high urinary Cu/Mo ratios on the day of VOR was negatively associated with ovarian stimulation and preimplantation IVF outcomes [12]. Together, this evidence reinforces that, for clinical application, the ratios of compounds with strong interactions may predict IVF outcomes better than the total concentrations of individual elements.

Zinc is the second most abundant trace element in the body [2,3]. Zinc is considered an essential element because it acts as a cofactor for more than 300 enzymes required for correct cell division, the synthesis and maintenance of genetic material, and cell metabolism [2]. The general population is mainly exposed to zinc through dietary sources, particularly meat products and shellfish [2,3]. However, different anthropogenic activities, such as mining and metallurgical operations, or handling products containing zinc, contribute to environmental zinc contamination [3,36].

Zinc is the most studied essential trace element in relation to IVF outcomes, although the evidence remains inconsistent across studies [9,10,11,12,20,21,22]. In our previous American cohort study [12], the zinc levels in plasma, FF, and urine were not related to IVF treatment outcomes. However, the Spanish cohort in this study demonstrated that higher levels of zinc in whole blood are associated with a lower oocyte yield following COS.

Although zinc is an essential element for human health, particularly reproduction [2], significant zinc exposure can have systemic repercussions, affecting the lungs, cardiovascular, gastrointestinal, and immune systems. Furthermore, an excessive body burden of zinc interferes with the metabolism of other essential trace elements, such as copper and iron [3,36].

Based on the relationship between copper and zinc being similar to what we previously described for copper and molybdenum, we incorporated the Cu/Zn ratio to evaluate whether it could become a more relevant clinical marker than the total levels of each element [37,38,39]. Corroborating the findings from our previous American cohort study [12], Spanish women with higher Cu/Zn ratios in whole blood responded better to ovarian stimulation, as evidenced by higher trigger day estradiol levels and oocyte yields. This association substantiates the importance of copper metabolism in reproductive success.

Lithium is an element that is naturally present in the human body, and it is fundamentally ingested through diet; however, human exposure to lithium has also been increased through the administration of psychotropic drugs [40,41,42]. Because of its involvement in biochemical reactions, its inclusion as an essential trace element remains controversial [40].

American patients undergoing IVF with higher lithium levels in FF responded poorly to ovarian stimulation and produced oocytes with a lower fertilization potential [12]. Spanish patients undergoing IVF with high levels of urinary lithium the morning of VOR had fewer embryos developing to the blastocyst stage, which corroborates the lithium-related decrease in oocyte competence and preimplantation embryo development described in cell and animal models [43,44]. Furthermore, lithium exposure was related to an increased miscarriage rate [12,45,46]. Together, these negative associations between lithium exposure and reproductive outcomes support the need to monitor environmental lithium exposure and evaluate the reprotoxicity of lithium-based drug treatment [47].

Besides participating in oxygen transport, iron is an enzymatic cofactor involved in energy production (e.g., cytochromes) and antioxidant defense mechanisms (e.g., catalase and peroxidase) [2]. Notably, this is the first study to report that women undergoing IVF with high urinary iron levels the day of VOR had a lower proportion of euploid embryos. However, most previous studies, including ours, did not find that FF and urinary iron levels had associations with IVF treatment outcomes [10,12,20]. Conversely, Rodriguez-Diaz et al. observed a correlation between higher iron concentrations in FF and decreased cleavage rates and poorer embryo quality [48]. The exact mechanism that may be behind these associations is unknown, although in vitro studies have related iron exposure with poorer oocyte quality [49]. We can only argue that the women in our Spanish cohort are in the age range in which the probability of oocyte aneuploidy is drastically increased [50], so they may be more sensitive to circulating iron levels than the women in the American cohort.

In the Spanish patients undergoing IVF, we did not observe significant associations between copper, manganese, and chromium concentrations and the IVF treatment outcomes evaluated. However, prior studies including our American cohort study identified associations that deserve to be mentioned.

Copper is an essential trace element that acts as a cofactor for numerous enzymes and neuropeptides [2,51,52]. In the general population, the main dietary sources of copper include shellfish, liver meat, grains, chocolate, and nuts [2]. In this study, there were no significant associations between the direct copper levels and reproductive outcomes following IVF and FET/SET, deviating from our evidence in American women undergoing IVF [12] and what other authors previously reported [9,21]. However, as described above, the relationships we observed with the Cu/Mo and Cu/Zn ratios suggest that copper might have a protective role regarding the ovaries. Further studies are required to determine whether the role of copper in estrogenic signaling [51,52] and oocyte quality [53,54] can be leveraged to improve fertility in subfertile women.

Manganese acts as a component of antioxidant enzymes and a cofactor of multiple enzymes, some of which are involved in steroidogenesis. Previous studies reported that women with high plasma and urinary manganese concentrations had better responses to ovarian stimulation and preimplantation IVF outcomes [9,12], and some authors have linked insufficient dietary intake with an increased risk of anovulation [55].

Although manganese levels were not comparable between biological fluids, whole blood manganese levels in the Spanish cohort were higher than those found in the plasma of American patients undergoing IVF [12]. This difference in concentration between the cohorts could explain the absence of an association in the Spanish population.

Finally, chromium is a metal which can have opposing effects on the body depending on its oxidation state [2]. Trivalent chromium acts as an essential trace element involved in glucose, carbohydrate, and lipid metabolism, whereas hexavalent chromium has been classified as a carcinogenic agent due to its ability to induce oxidative stress, DNA damage, chromosomal instability, and cell death [2]. Unfortunately, in our study, we were unable to differentiate between both oxidation states due to analytical issues. With the exception of our previous study finding that urinary chromium in American patients undergoing IVF were negatively correlated with the probability of a live birth [12], other studies failed to find significant associations between chromium concentrations in different biofluids with IVF treatment outcomes [9,27,56]. Given the antagonistic effects of its two oxidation states, in future studies, we will evaluate both chromium forms to differentiate between the two exposures.

Considering all of the geographical and sociocultural factors that may be influencing the contradicting associations observed between studies, besides the differences in age and BMI, we highlight diet as a leading confounding factor [3]. In future studies, taking into account the participants’ dietary patterns could provide deeper insights into this factor.

While there is current interest for adjuvant therapies to improve reproductive outcomes, such as micronutrient supplementation, in the context of assisted reproductive treatments, it has been reported that the best strategy for improving reproductive outcomes is adherence to the Mediterranean diet [57,58]. Part of this effect may be due to the fact that this diet is associated with higher concentrations of micronutrients [57]. In addition, the use of dehydroepiandrosterone, co-enzyme Q10, melatonin, and essential fatty acids may be beneficial in some situations [58]. Although it has been reported that subfertile women have low concentrations of essential trace elements [2,5,6,7,8] and that their supplementation may help to improve reproductive outcomes [5,7], in view of our results, this supplementation should only be prescribed in cases of deficiency of a particular trace element and monitored by a health care professional, as higher concentrations may be counterproductive to reproductive outcomes.

The negative associations related to essential trace element levels emphasize the importance of conducting biomonitoring studies in distinct communities. Environmental exposure does not seem to be generalizable as it rarely takes into account the full geographical and sociocultural context of the study population.

Finally, among this study’s limitations, we would like to highlight that this is a pilot study with a relatively small sample, and thus, the observations have restricted statistical power. Furthermore, this study did not account for the cumulative results from sequential ET cycles per participant or the effects of paternal exposure, which have been explored by other groups, or the interaction with the exposure to environmental pollutants [59,60,61,62,63].

Therefore, the next step would be to conduct studies in a larger cohort to simultaneously assess the impact of exposure to essential trace elements and other micronutrients on IVF treatment outcomes and their modifying effects on exposure to toxic compounds, such as non-essential trace elements, regarding IVF-related variables.

## 5. Conclusions

In conclusion, we found significant associations between essential trace elements and IVF treatment outcomes in Spanish women undergoing ICSI with PGT-A and SET/FET. Overall, our results show that, although trace elements may be beneficial for women seeking IVF treatment, an excess of some of these elements may be detrimental to their reproductive performance. While multicenter studies with larger cohorts are needed to confirm these results and delve deeper into the underlying biological mechanisms of how essential trace elements impact human reproduction, we recommend cautionary use of selenium supplementation in cases of deficiency to avoid overexposure. On the other hand, investigating how urinary molybdenum negatively affects clinical IVF outcomes may reveal new therapeutic targets. Finally, beginning to use the Cu/Zn and Cu/Mo ratios as more reliable predictors of clinical IVF outcomes than the total concentrations of copper, zinc, and molybdenum can help improve clinical decision making.

## Figures and Tables

**Table 1:** The baseline characteristics of the participating women (n = 51).

**Demographic Characteristics**	
Age (years), median [IQR]	39.00 [38.00, 41.00]
Body mass index (kg/m^2^), median [IQR]	22.97 [20.63, 25.12]
Race/ethnic group, n (%)	
- Hispanic–White	48 (94.1%)
- Afro-American	1 (2.0%)
- Hispanic	1 (2.0%)
- Other	1 (2.0%)
Education, n (%)	
- Elementary school	3 (6.5%)
- High school	5 (10.9%)
- Trade school	5 (10.9%)
- University	33 (71.7%)
Smoking, n (%)	
- Never smoked	23 (50.0%)
- Ex-smoker	13 (28.3%)
- Active smoker	9 (19.6%)
- Passive smoker	1 (2.2%)
**Reproductive Characteristics**	
Total FSH + hMG dose during COS (IU), median [IQR]	3300.00 [2437.50, 3925.00]
Trigger day E2 (pg/mL), median [IQR]	2083.00 [1622.50, 4045.50]
Number of retrieved oocytes, median [IQR]	12.00 [7.00, 16.00]
Maturation rate, % mean ± SD	80 ± 17%
Fertilization rate, % mean ± SD	75 ± 25%
Blastulation rate, % mean ± SD	54 ± 28%
Euploid rate, % mean ± SD	42 ± 34%
Transfer rate, n (%)	36 (70.6%)
Implantation (positive hCG) rate, n (%)	23 (63.9%)
Clinical pregnancy rate, n (%)	19 (52.8%)
Live birth rate, n (%)	17 (47.2%)
Goal rate, n (%)	17 (33.3%)

Note: the goal rate refers to the achievement of a live birth within a given cycle. COS, controlled ovarian stimulation; E2, estradiol; FSH, follicle-stimulating hormone; hCG, human chorionic gonadotropin; hMG, human menopausal gonadotropin; IQR, interquartile range; SD, standard deviation.

**Table 2:** The distributions of essential trace element concentrations in follicular fluid, whole blood, and urine obtained on the day of vaginal oocyte retrieval and in urine obtained on the day of embryo transfer.

	LOD	Samples below the LOD, n (%)	GM (SD)	20%	50%	80%
**Follicular Fluid (n = 29)**						
Copper (Cu) (ng/mL)	NA	0 (0%)	841.63 (300.01)	733.80	922.00	1120.92
Zinc (Zn) (ng/mL)	NA	0 (0%)	343.26 (115.35)	253.80	372.00	472.88
Copper/Zinc Ratio (Cu/Zn)			2.45 (0.89)	2.07	2.55	3.01
Molybdenum (Mo) (ng/mL)	1	2 (5%)	2.16 (1.74)	1.26	2.10	5.00
Copper/Molybdenum Ratio (Cu/Mo)			390.45 (314.53)	180.64	491.43	737.97
Selenium (Se) (ng/mL)	NA	0 (0%)	44.49 (15.44)	32.60	50.00	61.00
Lithium (Li) (ng/mL)	1	13 (33%)	1.33 (1.69)	0.50	1.40	3.62
Iron (Fe) (ng/mL)	NA	0 (0%)	694 (1661.39)	383.00	626.00	957.20
**Whole Blood (n = 40)**						
Copper (Cu) (ng/mL)	NA	0 (0%)	1039.42 (211.52)	893.44	1091.39	1240.97
Zinc (Zn) (ng/mL)	NA	0 (0%)	3614.45 (731.91)	3060.96	3640.56	4377.56
Copper/Zinc Ratio (Cu/Zn)			0.29 (0.07)	0.25	0.29	0.35
Molybdenum (Mo) (ng/mL)	NA	0 (0%)	3.84 (2.03)	3.38	3.95	4.44
Copper/Molybdenum Ratio (Cu/Mo)			272.76 (345.13)	218.51	276.12	320.32
Selenium (Se) (ng/mL)	NA	0 (0%)	91.09 (13.46)	80.94	90.85	99.37
Lithium (Li) (ng/mL)	NA	0 (0%)	6.56 (1.36)	6.16	6.77	7.43
Chromium (Cr) (ng/mL)	1.5	7 (15%)	2.74 (2.44)	1.75	3.04	4.86
Manganese (Mn) (ng/mL)	NA	0 (0%)	10.62 (6.67)	7.29	9.99	16.88
**Urine, VOR (n = 50)**						
Copper (Cu) (ng/mL)	NA	0 (0%)	11.67 (7.21)	7.27	12.34	19.55
Creatinine-corrected Copper (µg/g CR)			2.89 (1.81)	2.02	2.62	4.42
Zinc (Zn) (ng/mL)	NA	0 (0%)	310.1 (216.03)	186.67	356.93	532.58
Creatinine Corrected Zinc (µg/g CR)			75.35 (55.09)	42.36	82.82	130.14
Copper/Zinc Ratio (Cu/Zn)			0.04 (0.08)	0.02	0.03	0.07
Molybdenum (Mo) (ng/mL)	NA	0 (0%)	41.93 (34.77)	25.37	38.99	68.70
Creatinine Corrected Molybdenum (µg/g CR)			10.1 (4.93)	6.86	10.41	14.86
Copper/Molybdenum Ratio (Cu/Mo)			0.29 (0.28)	0.17	0.26	0.57
Selenium (Se) (ng/mL)	NA	0 (0%)	31.95 (10.49)	26.08	32.92	40.56
Creatinine Corrected Selenium (µg/g CR)			7.9 (2.86)	6.29	7.71	10.14
Lithium (Li) (ng/mL)	NA	0 (0%)	39.12 (31.75)	21.52	38.84	68.92
Creatinine Corrected Lithium (µg/g CR)			9.56 (9.19)	5.63	9.40	14.53
Iron (Fe) (ng/mL)	1	1 (2%)	7.87 (98.56)	5.00	5.00	13.88
Creatinine Corrected Iron (µg/g CR)			1.96 (38.71)	0.89	1.77	3.47
Chromium (Cr) (ng/mL)	0.5	28 (56%)	0.49 (1.06)	0.25	0.25	1.45
Creatinine Corrected Chromium (µg/g CR)			0.12 (0.33)	0.05	0.10	0.42
**Urine, Embryo Transfer Day (n = 27)**						
Copper (Cu) (ng/mL)	NA	0 (0%)	9.16 (67.83)	5.27	8.70	14.13
Creatinine Corrected Copper (µg/g CR)			2.83 (27.45)	1.36	2.40	3.68
Zinc (Zn) (ng/mL)	NA	0 (0%)	164.07 (188.63)	82.02	172.60	327.46
Creatinine Corrected Zinc (µg/g CR)			50.65 (68.6)	30.46	45.78	91.93
Copper/Zinc Ratio (Cu/Zn)			0.06 (0.44)	0.03	0.04	0.09
Molybdenum (Mo) (ng/mL)	NA	0 (0%)	33.18 (30.13)	19.54	28.43	57.12
Creatinine Corrected Molybdenum (µg/g CR)			10.24 (7.89)	6.72	10.25	15.54
Copper/Molybdenum Ratio (Cu/Mo)			0.28 (0.64)	0.13	0.28	0.46
Selenium (Se) (ng/mL)	NA	0 (0%)	26.08 (13.26)	17.67	22.66	43.01
Creatinine Corrected Selenium (µg/g CR)			8.05 (2.51)	6.31	7.76	10.41
Lithium (Li) (ng/mL)	NA	0 (0%)	29.12 (23.16)	15.00	29.15	54.80
Creatinine Corrected Lithium (µg/g CR)			8.99 (8.08)	6.15	9.41	13.90
Iron (Fe) (ng/mL)	5	5 (18%)	8.15 (10.45)	4.37	5.36	21.84
Creatinine Corrected Iron (µg/g CR)			2.51 (3.75)	1.20	2.34	4.58

Abbreviations: CR, creatinine; GM, geometric mean; LOD, limit of detection; NA, not applicable; SD, standard deviation; VOR, vaginal oocyte retrieval.

## Data Availability

The data presented in this study are openly available in Mendelei.

## Supplementary Materials

The following supporting information can be downloaded at https://www.mdpi.com/article/10.3390/cells13100839/s1. Figure S1: Spearman correlation matrix representing the relationships of each essential trace elements between biological matrices. Figure S2: Spearman correlation matrix representing the relationships between the essential trace elements within each biological matrix. Table S1: Mean differences (95% confidence interval) in ovarian response-related outcomes by essential trace element concentrations. Table S2: Mean differences (95% confidence interval) in the preimplantation IVF outcomes by essential trace element concentrations. Table S3: Odds Ratio (95% Confidence Interval) for the clinical reproductive variables.
