# Supplementary figures and images for: Higher Concentrations of Essential Trace Elements in Women Undergoing IVF May Be Associated with Poor Reproductive Outcomes Following Single Euploid Embryo Transfer
Source: Cells. 2024 May 15;13(10):839. doi: 10.3390/cells13100839 (PMC11119764; doi:10.3390/cells13100839)

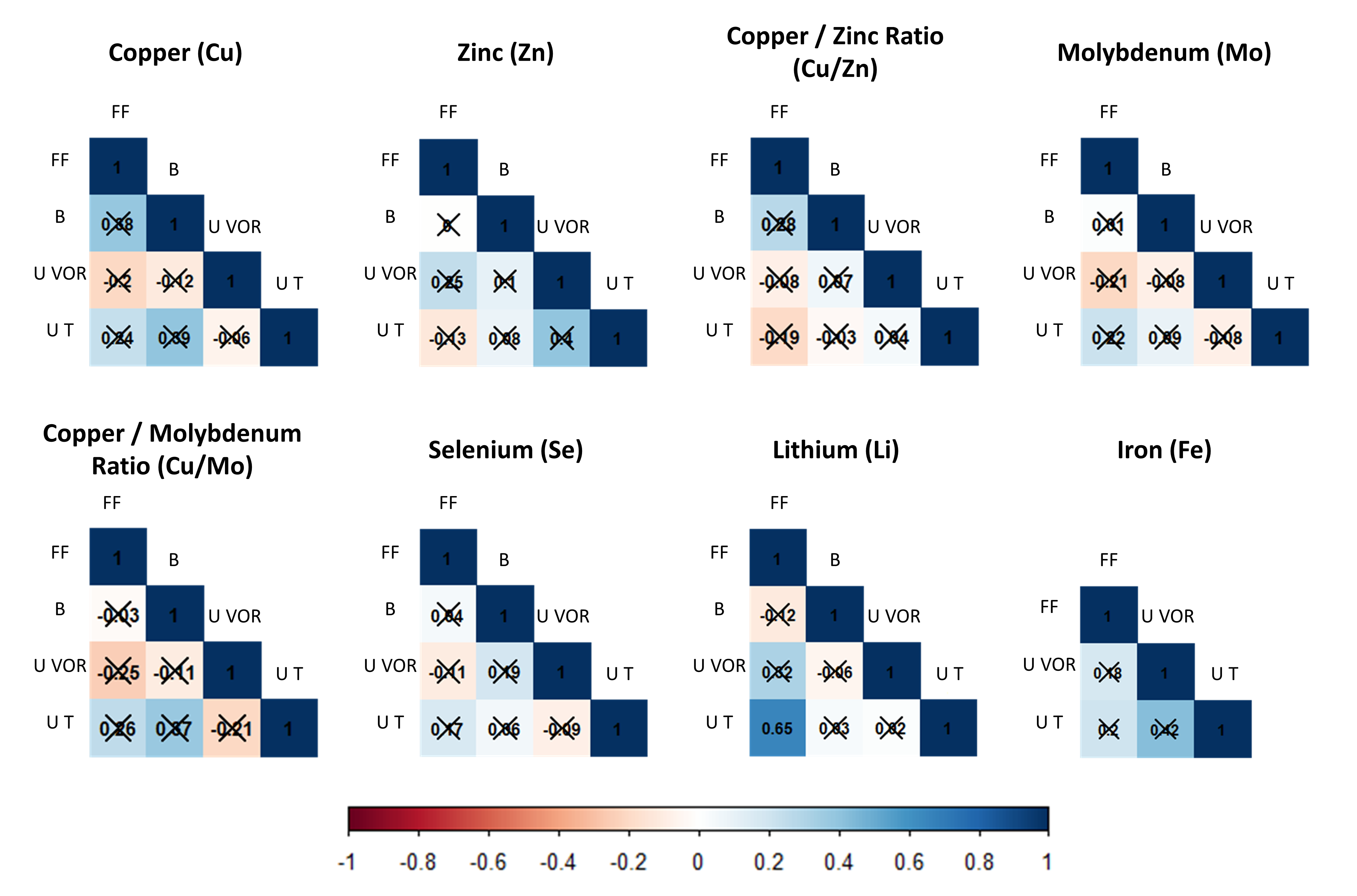

Supplement: Supplementary file 1 [file cells-13-00839-s001.zip › Fig S1 - Correlations metabolite Essential v3.tiff]

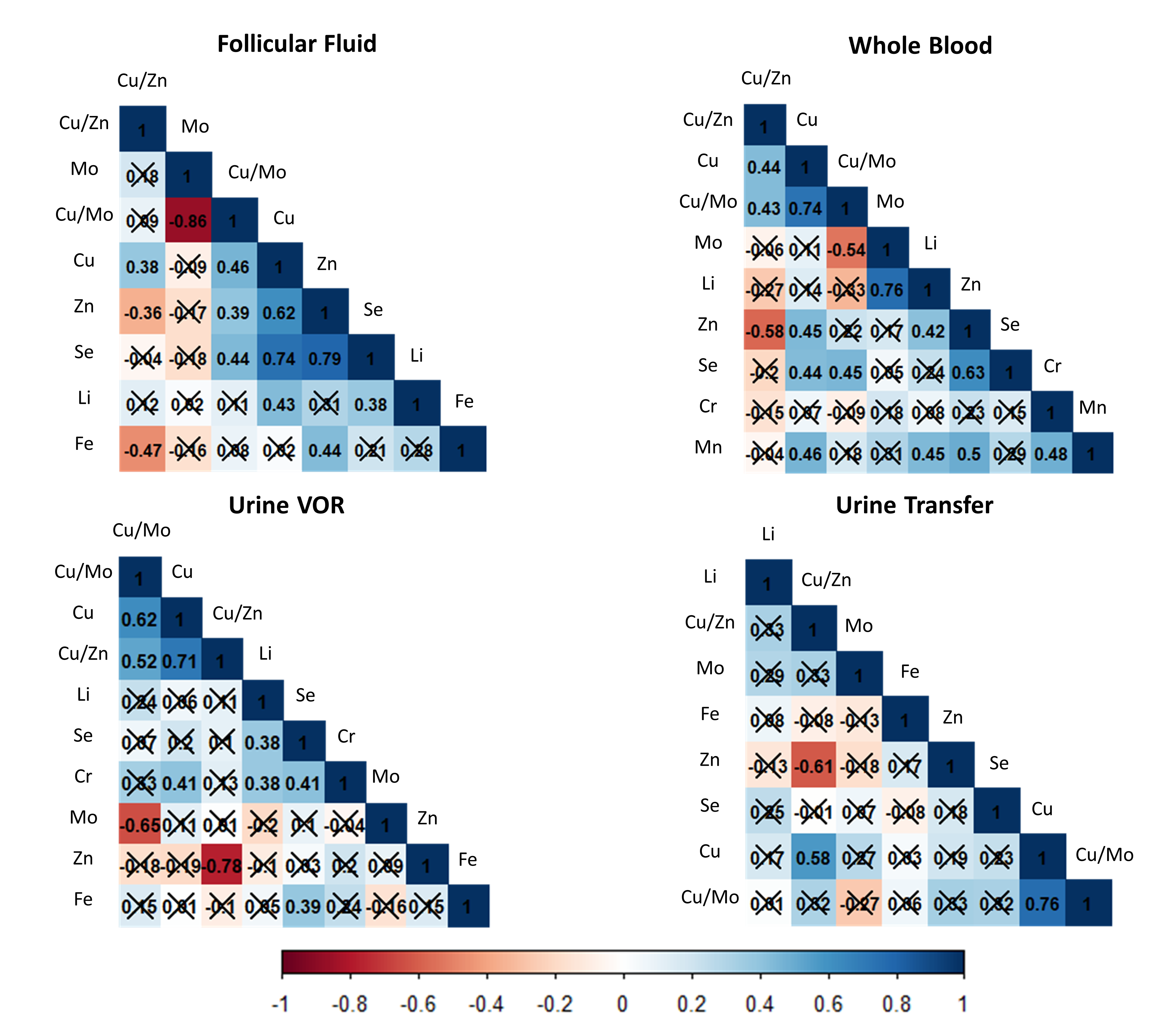

Supplement: Supplementary file 1 [file cells-13-00839-s001.zip › Fig S2 - Correlations matrix Essential v3.tiff]
